# Supplementary figures and images for: The organization of double-stranded RNA in the chikungunya virus replication organelle
Source: PLoS Negl Trop Dis. 2023 Jul 5;17(7):e0011404. doi: 10.1371/journal.pntd.0011404 (PMC10351700; doi:10.1371/journal.pntd.0011404)

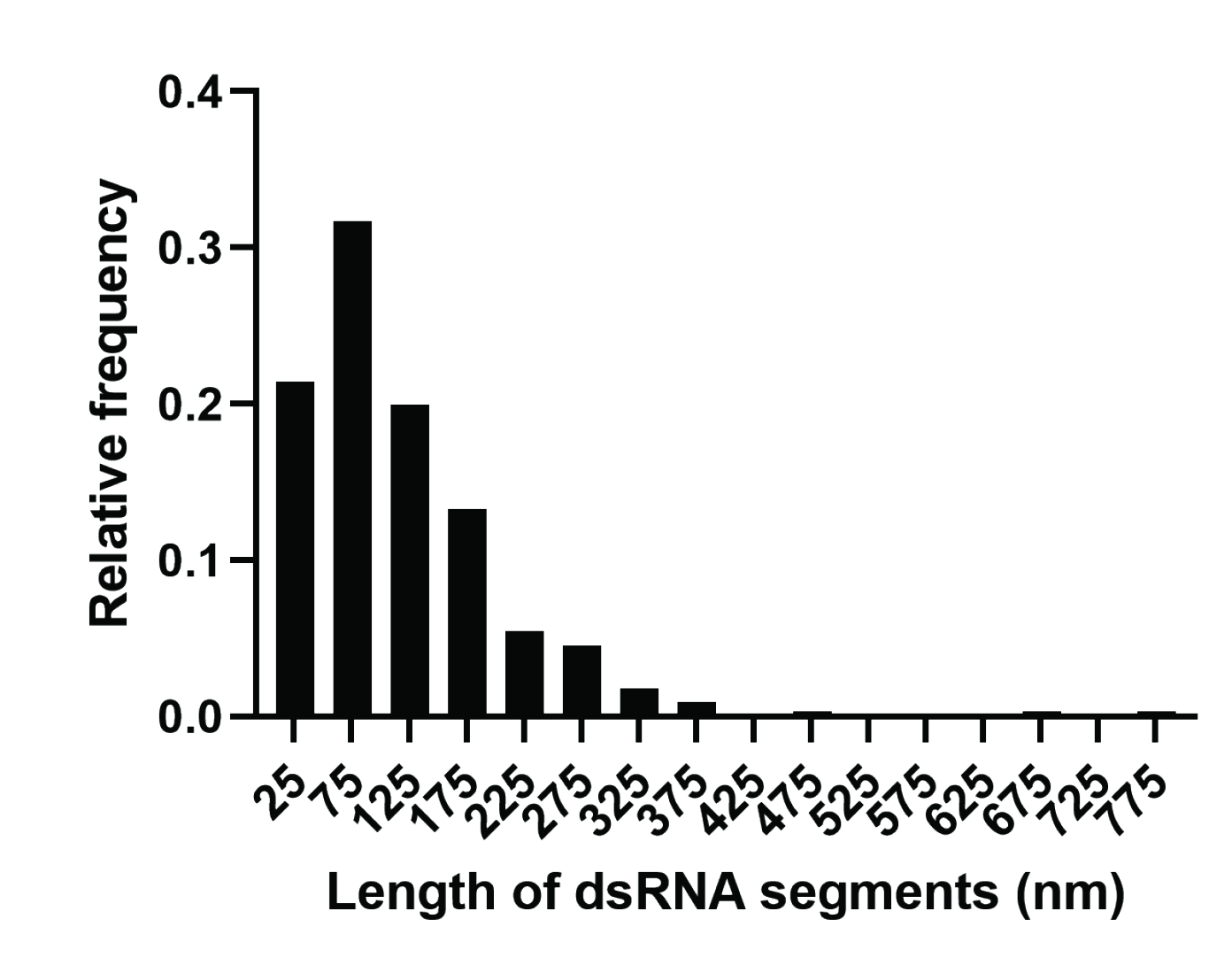

Supplement: S2 Fig — The histogram shows the distribution of lengths of uninterrupted filament fragments traced in spherules. The median value is 88.8 nm, corresponding to 347 base pairs. (TIF) [file pntd.0011404.s002.tif]

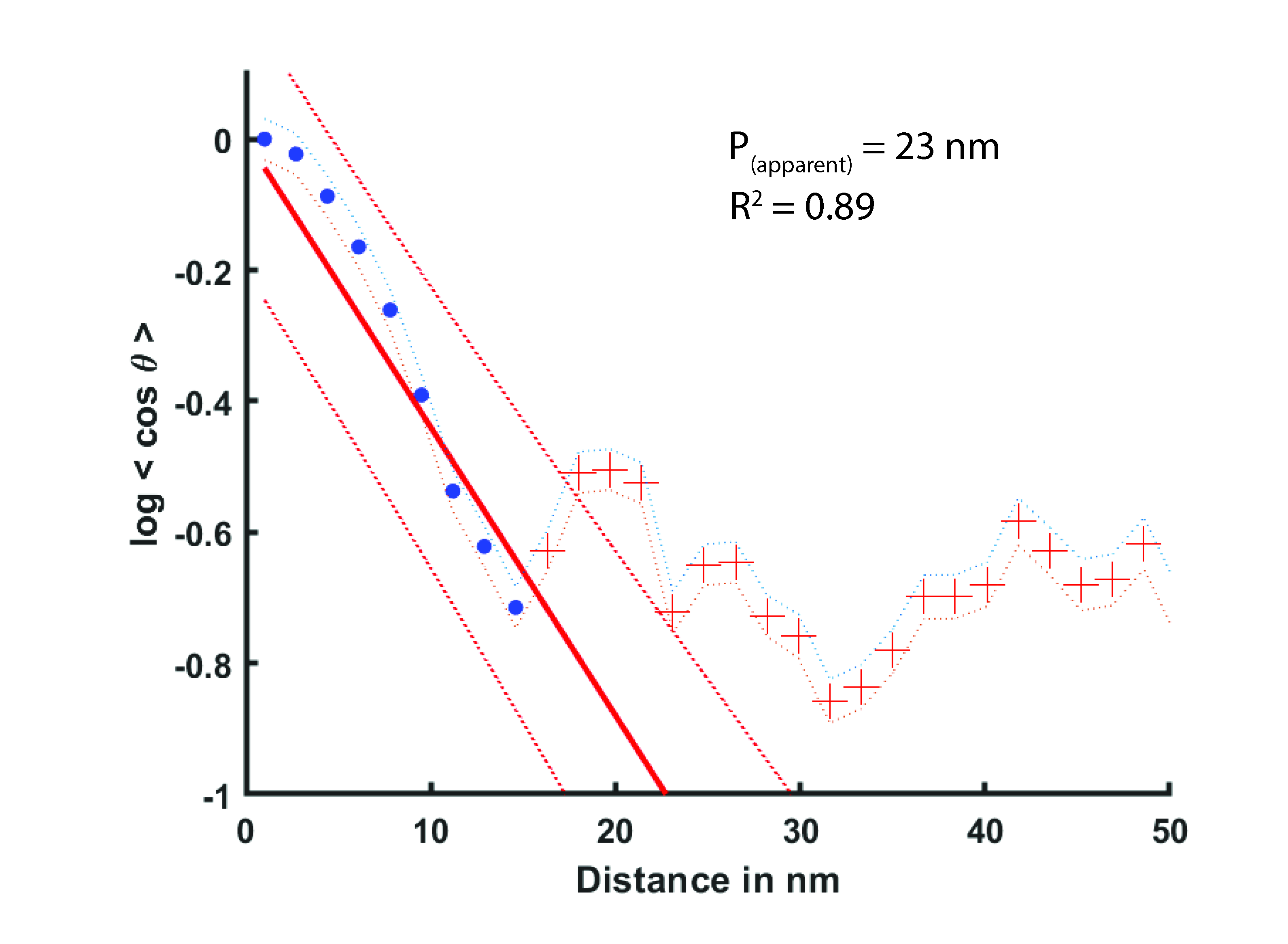

Supplement: S3 Fig — The outcome for one representative spherule of the estimation of the apparent persistence length of the dsRNA using the correlation decay between the cosines of tangents. Blue dots: linear portion of the curve. Red crosses: correlation values beyond the linear portion. Solid red line: extrapolation of the linear portion of the curve used to estimate the apparent persistence length. Dotted red lines: 95% confidence interval. (TIF) [file pntd.0011404.s003.tif]
