# Supplementary material for: The organization of double-stranded RNA in the chikungunya virus replication organelle
Source: PLoS Negl Trop Dis. 2023 Jul 5;17(7):e0011404. doi: 10.1371/journal.pntd.0011404 (PMC10351700; doi:10.1371/journal.pntd.0011404)
Supplement: Attachment — Submitted filename: response_to_reviewers_final.docx [file pntd.0011404.s005.docx]

**Dear Editor and Reviewers,**

**Thanks very much for the thorough and insightful review of our manuscript. Our point-by-point response is in bold below. We have also submitted one version of the revised manuscript with all changes highlighted in yellow.**

**Best wishes,**

**Lars-Anders Carlson and Timothée Laurent**

<b>Key Review Criteria Required for Acceptance?</b></br></br>
As you describe the new analyses required for acceptance, please consider the following:</br></br>

<b>Methods</b></br></br>
-Are the objectives of the study clearly articulated with a clear testable hypothesis stated?</br>
-Is the study design appropriate to address the stated objectives?</br>
-Is the population clearly described and appropriate for the hypothesis being tested?</br>
-Is the sample size sufficient to ensure adequate power to address the hypothesis being tested?</br>
-Were correct statistical analysis used to support conclusions?</br>
-Are there concerns about ethical or regulatory requirements being met?</br></br>

Reviewer #1: 1. The objectives of the study are clear with a testable hypothesis.
2. The study design is appropriated to address the objectives.
3. The population is clearly described.
4. The sample size is sufficient, specially given the technical limitations.
5. The statistical analysis is correct.
6. I do have a concern about the regulatory requirements. This based on the fact that the authors do not describe at all the methodology to induce the assembly of the vesicle in replicon expressing cells. There is no description of how the experiments were performed and in order to go from transfected or infected cells to Cryo-EM. Therefore, the methodology section must be improved.
**We thank you for the appreciation of our work. All analyses are based on data sets described in a previous publication (PMID: 36259931). The sample preparation including cell culture, the viral replicon particles system and plunge-freezing prior to imaging were described in detail in that paper. We have now also added a short summary of the sample preparation and data collection in the methods section (lines 113-119 of the revised manuscript), including a reference to the previous paper.**

Reviewer #2: In this research, the authors followed a well-designed methodological strategy to prove their hypothesis with sufficient veracity. The objectives of the work were appropriately described and proper scientific reasoning was followed to demonstrate their proposals about the assembly of the replication spherules of the Chikungunya virus, and its possible use for the design of more efficient RNA vaccines, with which the synthesis of proteins of vaccine interest is achieved for a better sensitization and activation of the immune system.
Based on the results of the investigation, they make correct statements about their proposal; some significant statements were:
1.      The viral protein nsP1 serves as a base for the assembly of a larger protein complex at the neck of the membrane bud; But in the absence of any negatively charged lipids, nsP1 did not bind appreciably to the vesicles; and nsP1 has concentration-dependent binding to PS-containing membranes.
2.      Spherules contain a single copy of the viral genome in double-stranded form.
3.      The energy released by RNA polymerization is found to be sufficient to remodel the membrane to the characteristic spherule shape.
4.      Alphaviruses are not only a major source of morbidity, but their unique RNA replication mechanism is also used to develop self-replicating RNA vaccines that induce a more potent immune response than conventional mRNA vaccines

**We thank you for your feedback.**

Reviewer #3: No major experiments/analyses are required for this study.

--------------------

<b>Results</b></br></br>
-Does the analysis presented match the analysis plan?</br>
-Are the results clearly and completely presented?</br>
-Are the figures (Tables, Images) of sufficient quality for clarity?</br></br>

Reviewer #1: 1. The analysis match the plan.
2.1 The results are not always clearly presented and some discussion seem incomplete. In particular, it is not clear what do the class averages in figure 1C-G correspond in a vesicle like the one on figure 1A. Also, it is not clear if the apparent persistence length depends on the size of the vesicle. Please clarify these problems.

**These are indeed important points to discuss. Due to the clear membrane density, we can unambiguously state that class C in figure 1 is the dsRNA curving along the membrane. For the other classes (Fig. 1D – G) we can only say that they are likely not membrane-apposed since the dsRNA is homogeneously dispersed in the spherule (Fig. 2C), and most dsRNA is hence at a larger distance from the membrane. We have modified the text (lines 255-257 in the revised manuscript) to add this consideration.**

**We think that the apparent persistence length would correlate with the vesicle size if the variation in vesicle size was larger. But when we plotted the persistence length obtained for individual spherules against their respective volumes there was no significant correlation with our limited sample size. We thus decided to not include it in the revised article, but we show the diagram below for reference (note that the axes don’t start at 0):**

2.2 Interestingly they found a P < 63 nm. However, when they calculate ro zero and correlate that with the phase diagram they use P = 63 nm. I would suggest to repeat this calculation with P = 25-32 nm, I am sure this P will result in a scenario that agrees with your results.

**We considered this suggestion of the reviewer, but according to our understanding of the theoretical model by Liang et al it would be mathematically wrong to use the apparent persistence length (22 nm). However, it is of course possible that the true persistence length is closer to our measured Papparent than the previously published 63 nm. We have amended the text to account for this possibility (lines 342-345 in the revised manuscript).**

2.3 In the discussion they claim that P = 23 might be an underestimation. However, this might not be true. For example Cifra et al 2008 JPB showed that the persistence length of a worm-like polymer under confinement changes as a function of the confinement under certain stiffness regimes. They predicted that for such polymer you should see a toroidal structure as you propose in figure 3b. The fact that you do not observe that structure might suggest that P is lower than expected because the RNA inside is not 100% ds. In fact, based on Cifra´s work P should increase for a worm-like polymer, hence the RNA inside the spheres might be more complex than expected. I would suggest to take this in consideration to analyze the results.

**Thank you for your suggestion. Firstly, our system slightly differs from what is reported in Cifra’s work. A spherule is not a rigid structure since its membrane can expand and contract in response to pressure excerted by the luminal RNA. Cifra et al. report that the persistence length of polymers increases in a cylindrical confinement and decreases in a spherical confinement as pointed out in table 1 of their article.**

**In the light of your comment, we now realize that our original figure 3B was not well constructed. We did not intend to show a toroid but instead, in a very simplistic manner, to suggest that the dsRNA has no preferred orientation with respect to the neck complex (this can for instance happen due to a random packaging). We have now modified the figure to hopefully look more like a random packaging, and also changed the figure legend (line 286) to reflect this.**

2.4 it is possible that the dsRNA is bound to proteins that could condense it and thus lower the persistence length? Also, please discuss more the possibility that the RNA is not completely ds.

**You are right. It is very likely that the dsRNA and/or segments of ssRNA are associated with proteins, and they may reduce its apparent persistence length. It is equally possible that interspersed ssRNA regions have the same effect. In the revised manuscript we mention the possibility that this may alter the persistence length (lines 313-317).**

2.5 At some point the mentioned 80% of the RNA is present as dsRNA, but this is not consistent with table 1. Please clarify.

**It is correct that ~80% of the genome was traced. Then these 80% were subjected to subtomogram averaging. This method produced averages (shown in Fig. 1C-G), in which the consensus structure of the RNA appears, whereas parts that are variable between the individual pieces disappear. That is where we “lose” another 27%. Table 1 indicates the percentage of the genome that was present in the consensus structure of each class, totalling ~52%. We have now added one more row in the table (line 227) indicating the sum of the dsRNA present in our averages. We have modified the table legend (lines 223-225) to make it unambiguous. We have also edited figure 1 H to clearly state that 27.3% of the dsRNA was not averaged and 20.3% of the dsRNA was not present in our initial tracing.**

2.6 The statements in lines 215-218. On the one hand, they are right that there no single conformation. On the other hand, they got the answer wrong; dsDNA is statisical polymers with an ensemble of conformations. Hence, just the nature of dsRNA is enough to not have a single conformation.
**Thanks for pointing this out. Our aim was to contrast the spherules with virions, in which dsRNA is indeed frequently found in a single conformation (since the capsid generates a single energy minimum conformation). Hence, a statistical polymer model with multiple conformations is not a good model for some types of capsid-bound viral nucleic acid. But the dsRNA in a spherules is only constrained to the membrane neck at a single point, and is continuously passed through this point, so the statistical polymer method is better suited. We changed the text to describe this (lines 261-265 in the revised manuscript).**

Reviewer #2: The results described in the manuscript are relevant and original, since they not only describe the structure and stability of the replication spherules of this virus, which is important to clarify the replication mechanisms of this virus, but also open the possibility of its use as a gene expression strategy of interest in vaccines and other therapeutic strategies.
As mentioned above, the experimental strategy was carefully designed; perhaps in the study of the participation of phospholipids in the binding of NSP1 to the membrane, some experiment of colocalization of NSPs proteins with antibodies could be included to ensure that NSP1 is the anchor for other NSPs. The colocalization study would only serve to confirm how NSP1 participates in the union of the other NSPs in the organization of the spherules and its possible role in viral replication.

**This is an interesting suggestion but outside the scope of the current study. It was partially dealt with in the previous paper of ours (Laurent et al, eLife 2022), and work by a competing group (PMID: 36449616).**

Reviewer #3: Most results are clearly represented, and figures are of sufficient quality.

**We thank you for your appreciation of our work.**

--------------------

<b>Conclusions</b></br></br>
-Are the conclusions supported by the data presented?</br>
-Are the limitations of analysis clearly described?</br>
-Do the authors discuss how these data can be helpful to advance our understanding of the topic under study?</br>
-Is public health relevance addressed?</br></br>

Reviewer #1: 1. the conclusions are partially supported by the data presented. I would suggest to also analyze how the distribution of the RNA changes by repeating the same calculation but with P = 23 nm. Perhaps, this calculations will result in regime that is consistent with the data (it could lower the amount of estimated dsRNA in line 280).

**Thanks for this suggestion, please see our response to the same consideration above (2.2).**

2. The limitations of the method with respect to the measurements are not clearly addresses. This might be because the group is an expert in these techniques but it would benefit the reader to have such discussions.

**Indeed a good point, and we now have a discussion of this as part of the final paragraph of the discussion (lines 352-359).**

3. Given the scope of the journal it would be good to discuss how these data can be helpful to advance our understanding of the topic under study and its relevance in public health.

**Thank you for pointing this out. We have now added an outlook to the end of our discussion (lines 356-359).**

Reviewer #2: The authors show a scientific dialogue with previous studies and are supported by results that they have previously described. It is important to mention that tomography coupled with microscopy has contributed to an advance in the knowledge of this area of research.
The research shows a substantial advance in the knowledge of viral replication spherules and their possible application in the development of vaccines and other therapeutic strategies such as the expression of genes that regulate certain metabolic pathways or silencing RNAs.

**We thank you for your feedback**

Reviewer #3: Discussions and conclusions are supported mainly by the data. However, their contents seem too technical and specific, making it difficult for the general reader to understand.
**Thank you for your feedback, we have now added an outlook in our discussion to put our research in perspective, highlighting key elements for the general audience (lines 352-359.**
--------------------

<b>Editorial and Data Presentation Modifications?</b></br><br/>
Use  this section for editorial suggestions as well as relatively minor modifications of existing data that would enhance clarity. If the only modifications needed are minor and/or editorial, you may wish to recommend “Minor Revision” or “Accept”.

Reviewer #1: The editorial and data presentation modifications are minimal. It would be better go have a detailed methodology of the work with cells, how some of the data was treated. Most of my comments are in the previous sections.

Reviewer #2: For all of the aforementioned, I recommend that the manuscript be accepted.

Reviewer #3: N/A

--------------------

<b>Summary and General Comments</b></br></br>
Use  this section to provide overall comments, discuss strengths/weaknesses of the study, novelty, significance, general execution and scholarship. You may also include additional comments for the author, including concerns about dual publication, research ethics, or publication ethics. If requesting major revision, please articulate the new experiments that are needed.

Reviewer #1: The strength of this article is the high quality of the data and the careful analysis to understand the physical properties of dsRNA inside the replication vesicles. To the best of my knowledge this is a novel research that could inspire theoretical physicist to re-evaluate classical models.
I have requested a minor revision because my observations have be easily addressed.

**We thank you for your valuable feedback and have now addressed your concerns**.

Reviewer #2: No comment

Reviewer #3: In this manuscript, Laurent and Carlson extended their previous Cryo-EM study of the CHIKV spherules and provided some new insights into the structural organization of CHIKV dsRNA in the spherules. Although the spherules the authors analyzed were not the authentic ones formed in CHIKV-infected cells, perhaps future work will pick up this slack through the use of infectious viruses. Particularly, it will be intriguing to know how the 26S subgeneric RNA encoding structural proteins are organized.
**This is an interesting and important point. We would be just as intrigued as the reviewer, and hope that we can address this in future studies through work with infectious virus instead of viral replicon particles.**

The manuscript is well-written and would be acceptable in its current form. However, it can be strengthened by discussing whether different conformations of dsRNA (described in Figures 1 and 3) would be functionally different in terms of the single-stranded RNA synthesis and biology of CHIKV.

**Thank you for your feedback. We don’t have any such insights from the current study but mention this exciting prospect in the final paragraph of the revised discussion (lines 354-356).**
